# Discovery of Electrophiles and Profiling of Enzyme Cofactors

**DOI:** 10.1002/cpch.86

**Published:** 2020-11-16

**Authors:** Suzanne E. Dettling, Mina Ahmadi, Zongtao Lin, Lin He, Megan L. Matthews

**Affiliations:** ^1^ Department of Chemistry University of Pennsylvania Philadelphia Pennsylvania; ^2^ Zenagem LLC Philadelphia Pennsylvania; ^3^ Biochemistry and Molecular Biophysics Graduate Group, Perelman School of Medicine University of Pennsylvania Philadelphia Pennsylvania

**Keywords:** chemoproteomics, de novo PTM discovery, enzyme activity, post‐translational modifications, reverse‐polarity activity‐based protein profiling

## Abstract

Reverse‐polarity activity‐based protein profiling (RP‐ABPP) is a chemical proteomics approach that uses nucleophilic probes amenable to “click” chemistry deployed into living cells in culture to capture, immunoprecipitate, and identify protein‐bound electrophiles. RP‐ABPP is used to characterize the structure and function of reactive electrophilic post‐translational modifications (PTMs) and the proteins harboring them, which may uncover unknown or novel functions. RP‐ABPP has demonstrated utility as a versatile method to monitor the metabolic regulation of electrophilic cofactors, using a pyruvoyl cofactor in *S*‐adenosyl‐l‐methionine decarboxylase (AMD1), and to discover novel types of electrophilic modifications on proteins in human cells, such as the glyoxylyl modification on secernin‐3 (SCRN3). These cofactors cannot be predicted by sequence, and therefore this area is relatively undeveloped. RP‐ABPP is the only global, unbiased approach to discover such electrophiles. Here, we describe the utility of these experiments and provide a detailed protocol for de novo discovery, quantitation, and global profiling of electrophilic functionality of proteins. © 2020 The Authors.

**Basic Protocol 1**: Identification and quantification of probe‐reactive proteins

**Basic Protocol 2**: Characterization of the site of probe labeling

**Basic Protocol 3**: Determination and quantitation of electrophile structure

## INTRODUCTION

Post‐translational modifications (PTMs) are covalent alterations of a protein that take place on one or more encoded amino acids. Major aspects of protein function, structure, and regulation (e.g., protein‐protein interactions, protein stability, turnover, localization of proteins, etc.) are often mediated by PTMs, contributing to diversification of the proteome (Walsh, Garneau‐Tsodikova, & Gatto, 2005). Nearly all mature proteins undergo PTMs, and they are context dependent, meaning that they can be activated and inactivated to meet the immediate demands of the cell.

**Figure 1:**
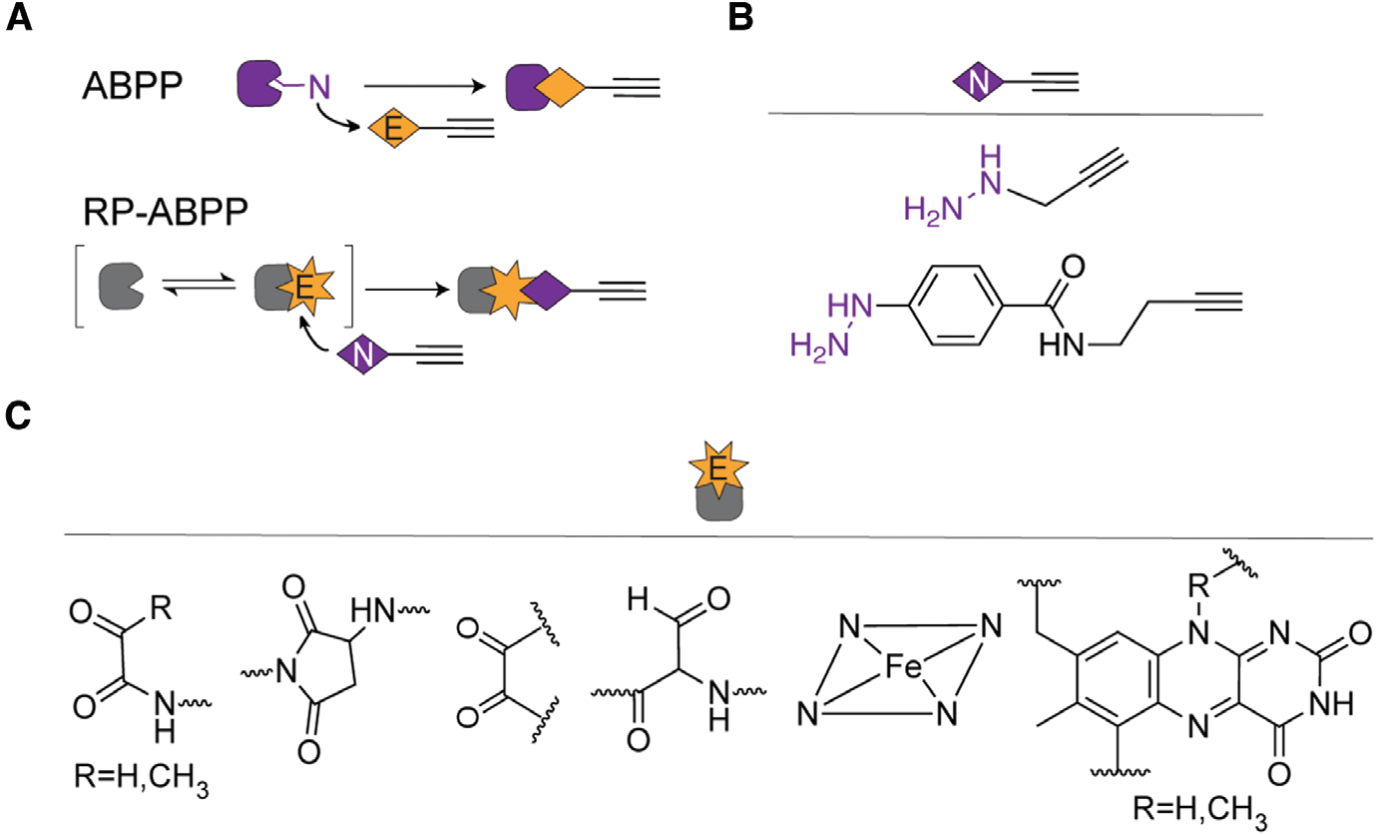
ABPP and RP‐ABPP. (**A**) Schematic of ABPP and its reverse‐polarity counterpart. “N” represents nucleophilic reactivity on an amino acid and “E” represents an electrophile probe. In the RP‐ABPP schematic, “E” represents an electrophile PTM and “N” represents a nucleophile probe. (**B**) Structures of hydrazine probes used previously (Matthews et al., 2017). (**C**) Enzyme cofactors known to react with hydrazines (Augusto et al., 1982; Binda et al., 2008; Carlson et al., 2008; Klaene et al., 2014; Shantz et al., 1992).

Traditionally, most studies of PTMs have been carried out in vitro in a case‐by‐case manner (e.g., using radioactive isotope‐labeled substrates or western blot analysis). However, the complexity and diversity of PTMs force researchers to utilize high‐throughput bioanalytical approaches in native biological systems to accelerate their identification and characterization. Moreover, because of the low abundance of many modified proteins, efficient purification methods and sensitive detection are necessary to study PTMs. During the past decade, mass spectrometry coupled with upstream purification and enrichment steps have paved the way for both targeted and unbiased screening of the proteome, and specifically of PTMs in native biological systems. These methods rely on efficient enrichment steps that target and pull out desired PTMs from complex biologic samples. The enrichment techniques developed are based on protein‐protein interaction (such as antibodies to capture the protein of interest) or protein‐substrate interaction (such as chemical reporters carrying an unnatural alkyne handle). A recently developed new chemical proteomics technology, reverse‐polarity activity‐based protein profiling (RP‐ABPP), utilizes nucleophilic probes to capture reactive electrophile PTMs in cells, thus allowing global discovery of electrophilic functionality in proteins (Fig. 1A; Matthews et al., 2017). RP‐ABPP was developed from activity‐based protein profiling (ABPP), a related chemical proteomics method that characterizes the nucleophilic reactivity of amino acids in the proteome by utilizing electrophilic probes to target functional nucleophiles (Fig. 1A; Cravatt, Wright, & Kozarich, 2008).

RP‐ABPP is the first, and so far only, global, unbiased method that uses nucleophilic hydrazine probes to capture the most reactive electrophiles present in the cell (Fig. 1B). Hydrazines were selected as the nucleophilic group due to their known ability to react with, and inhibit, oxidative and electrophilic cofactors (Fig. 1C) including pyruvoyl cofactors (Shantz, Stanley, Secrist, & Pegg, 1992), aspartimide sites (Klaene, Ni, Alfaro, & Zhou, 2014), quinone cofactors (Klinman & Bonnot, 2014), and formylglycyl cofactors (Carlson et al., 2008), as well as protein‐bound heme (Augusto, Kunze, & de Montellano, 1982) and flavin cofactors (Binda et al., 2008). Additionally, hydrazines exhibit high nucleophilicity and low basicity due to the alpha effect. These hydrazine probes are deployed in living cells to focus the results on electrophiles that are functional and strongly reactive in an endogenous setting.

Initial profiling by this unbiased screen demonstrated that there are functional and active electrophiles incorporated onto proteins post‐translationally in the cell (Matthews et al., 2017). Previous applications of RP‐ABPP have demonstrated electrophilic activity on numerous disease‐associated proteins [e.g., Kelch‐like ECH‐associated protein 1 (KEAP1) in cancer, amyloid precursor protein (APP) in Alzheimer's disease, fat mass and obesity‐associated protein (FTO) in obesity, etc.] that were not previously known to harbor such reactivity (Matthews et al., 2017). RP‐ABPP can be used for discovery of previously unknown PTMs, as was done in the discovery of the glyoxylyl modification on SCRN3 (Matthews et al., 2017). Additionally, RP‐ABPP also shows potential in profiling and monitoring known electrophilic cofactors, as was shown for the pyruvoyl cofactor of *S*‐adenosyl‐l‐methionine decarboxylase (AMD1). These experiments were performed using RP‐ABPP in two human cell lines, but the methods can also be applied in living organisms ranging from human pathogens to mouse models. Characterizing the structure and function of electrophile PTMs and the proteins that harbor them can elucidate unknown or novel functions of proteins in an endogenous setting, thereby improving our understanding of disease mechanisms and potentially even revealing new drug targets. Further, because the probes are potent inhibitors as well as discovery tools, they may eventually serve as a launching point for the development of selective small‐molecule inhibitors with pharmacological properties.

**Figure 2:**
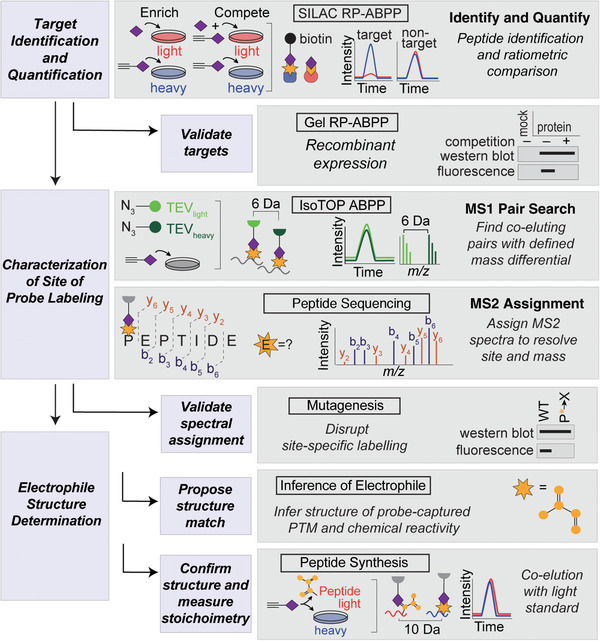
Schematic flowchart of RP‐ABPP experiments. Heavy (H) and light (L) cells, proteomes, and peptides are depicted in blue and red, respectively. Target identification and quantification is performed using SILAC RP‐ABPP extracted parent ion chromatograms and corresponding H/L ratios for tryptic peptides of probe targets quantified in enrichment and competition experiments. Targets are validated by gel RP‐ABPP western blots and RP‐ABPP data for hydrazine probe–treated transfected cells expressing a protein target. The first lane corresponds to a control transfection (“mock”) with the appropriate empty expression vector. The site of probe labeling is characterized using isoTOP ABPP experiments to determine coeluting isotopically differentiated peptide pairs and sequencing the ions to resolve the modified site. The site of probe labeling is validated through comparison of mutation and wild‐type (WT) probe‐labeling and expression profiles. The electrophile is determined, confirmed, and stoichiometrically quantified by inferring the electrophile and coelution of heavy‐Arg/Lys‐labeled transfected cells treated with probe (followed by processing by isoTOP‐ABPP) with a light‐amino‐acid‐labeled standard.

There are three major aims of the general workflow: (1) identifying and quantifying proteins with probe reactivity from native biological systems, (2) specifying the site where the electrophile is located on the protein and reacted with probe, and (3) determining the structure of the electrophile prior to probe capture. These aims are accomplished through a series of experiments illustrated in Figure 2. These experiments utilize a chemical probe, containing a nucleophilic hydrazine warhead and an alkyne handle for detection and enrichment via “click” chemistry [Cu(I)‐catalyzed azide‐alkyne cycloaddition (CuAAC)] to azide reporter tags. For downstream analysis via mass spectrometry, a biotin‐azide tag is used to enable the enrichment of probe‐bound targets on streptavidin resin. Conversely, for gel‐based detection, a rhodamine‐azide tag is used to visualize the bound electrophile using in‐gel fluorescence. To identify and quantify protein targets (Basic Protocol 1), enrichment and competition experiments are performed in stable isotope labeling by/with amino acids in cell culture (SILAC) RP‐ABPP followed by multidimensional liquid chromatography‐tandem mass spectrometry (LC‐MS/MS). The targets are then validated through recombinant expression and in‐gel RP‐ABPP. Determination of the site of labeling (Basic Protocol 2) is achieved through the use of isotopic tandem orthogonal proteolysis (isoTOP) ABPP, and the residue(s) harboring the PTM is identified by a hybrid sequencing approach followed by mutagenic analysis to confirm the finding. Finally, the structure of the electrophile is determined, confirmed, and stoichiometrically quantified by coelution with a synthetic peptide standard (Basic Protocol 3). This article provides an overview of the basic workflow to identify and characterize the electrophilic functionality of proteins using these protocols.

## Basic Protocol 1 IDENTIFICATION AND QUANTIFICATION OF PROBE‐REACTIVE PROTEINS

The first aim of RP‐ABPP methodology is to determine probe‐reactive proteins and quantify their reactivity. Probe‐reactive proteins are identified and quantified through probe treatment in live cells followed by lysis, conjugation of labeled proteins to a reporter tag (biotin‐azide), enrichment using streptavidin beads and quantitative mass spectrometry (MS) analysis, and validation via recombinant expression protocols as described in Matthews et al. (2017). Quantitation of this reactivity allows the exclusion of weakly reactive or low‐stoichiometry electrophiles to bias toward functional sites. This strategy allows the further pursuit of highly reactive target proteins that have a high likelihood of containing functional electrophiles.

RP‐ABPP experiments begin with treatment of cells with probe, competitor, or probe and competitor mixture, which is followed by proteome harvesting and enrichment. The probe design includes a reactive nucleophile, linker/scaffolds, and an alkyne handle. The competitor is the analog of the probe without the alkyne handle and therefore is not amenable to click chemistry (“non‐clickable”). These steps generate labeled soluble and membrane proteomes from the cell culture at a known concentration, which can be used as the starting material for the majority of downstream RP‐ABPP experiments. To bias this approach toward targets with near‐complete reactivity with the probe and avoid lower‐stoichiometry adducts, both enrichment and competition are necessary. Both experiments are achievable using SILAC methodology in which cells are grown in medium containing either natural‐abundance lysine and arginine (“light” medium) or ^13^C‐ and ^15^N‐enriched amino acid isotopologues (“heavy” medium).

**Figure 3:**
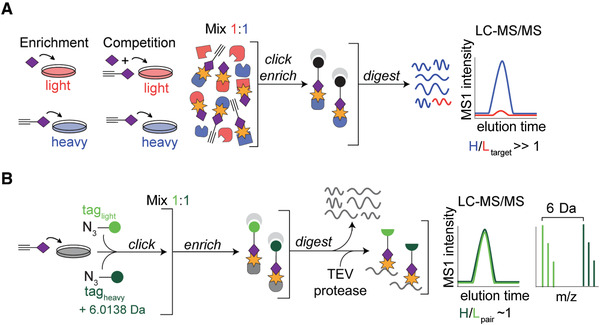
Schematics for characterization of protein targets and their sites of probe labeling. (**A**) Schematic for MS‐based quantitative (SILAC) proteomics experiments (enrichment and competition) as described in the text. Heavy (H) and light (L) cells, proteomes, and peptides are depicted in blue and red, respectively. (**B**) Characterization of probe‐labeled peptides using the isoTOP‐ABPP method as described in the text. Heavy and light tagged proteins and peptides are depicted in dark and light green, respectively.

SILAC can be utilized in RP‐ABPP enrichment and competition experiments, as shown in Figure 3A. In enrichment experiments, heavy cells are treated with probe and light cells with non‐clickable competitor to serve as control. In competition experiments, heavy cells are treated the same; however, light cells are treated with probe and an excess amount of competitor (10×). Enrichment and competition can also be shown using gel experiments. For MS‐based analysis, labeled proteomes are conjugated to biotin‐azide tags via click chemistry and enriched on streptavidin resin to remove unlabeled proteins. Proteins are then digested and analyzed via LC‐MS/MS. As the proteins incorporate the amino acids from the SILAC media (heavy or light), peptides will contain a known mass shift. That defined mass shift is extractable via mass spectrometry to quantify differences in the abundance of peptides in the respective proteome by ratiometric comparison. Coelution of isotopically differentiated peptides is a common strategy in RP‐ABPP, as will be demonstrated throughout this paper. Proteins with high heavy‐to‐light ratios for competition and enrichment are deemed to be robust high‐reactivity protein targets, and the reactivity is quantified using these ratios (Fig. 3A). Such profiling experiments were adapted from advanced protocols with electrophilic probes (Adibekian et al., 2011; Hulce, Cognetta, Niphakis, Tully, & Cravatt, 2013; Martin, Wang, Adibekian, Tully, & Cravatt, 2012; Niphakis et al., 2015).

Once high‐reactivity targets have been determined, they can be validated by demonstrating hydrazine reactivity as an intrinsic property of the protein targets shared by both the endogenous and recombinant forms of these proteins. This validation is performed by treatment of transfected cells with probe, followed by conjugation to azide‐rhodamine, and visualization of a strong fluorescent band at the appropriate molecular weight. This band should be absent in “mock”‐transfected cells treated with excess non‐clickable agents as this should block probe labeling of each protein. In addition, recombinant expression of each protein and lack of expression in “mock”‐transfected control cells are confirmed by western blotting. In order to perform these transfections, target genes need to be obtained in mammalian expression vectors. These vectors can be purchased or made using cloning protocols as previously described (Matthews et al., 2017).

### Materials

Hydrazinium chloride salts of probes and competitors (non‐clickable analogs), synthesized in house (Matthews et al., 2017)
Dimethyl sulfoxide (DMSO; Sigma‐Aldrich, cat. no. D5879)
Sodium hydroxide (Fisher Scientific, cat. no. S318‐500)
Colorphast pH indicator strips from pH 5 to 10 with accuracy of 0.5 pH unit (Millipore Sigma)
Low‐passage human embryonic kidney (HEK293T) and human breast cancer (MDA‐MB‐231) adherent cell lines (ATCC)
Dulbecco's Modified Eagle Medium (DMEM; high glucose, ll‐glutamine, pyruvate; Gibco, cat. no. 11995065)
Fetal bovine serum (FBS; Corning, cat. no. MT35‐010‐CV)
Penicillin/Streptomycin Antibiotic/Antimycotic (Gibco, cat. no. 15240062)
Dulbecco's phosphate‐buffered saline (PBS), pH 7.4 (Invitrogen, cat. no. 14040‐133)
10 mM Na‐HEPES buffer, pH 7.5 (Fisher Scientific, cat. no. BP3104)
DC (detergent‐compatible assay) protein assay kit (Bio‐Rad, cat. no. 5000111)
DMEM for SILAC (lysine and arginine free; ThermoFisher Scientific, cat. no. 88364)
Dialyzed fetal bovine serum (dFBS; Silantes, cat. no. 281001200)
l‐[^13^C_6_^15^N_2_]lysine hydrochloride (Sigma‐Aldrich, cat. no. 608041)
l‐[^13^C_6_^15^N_4_]arginine hydrochloride (Sigma‐Aldrich, cat. no. 608033)
l‐Lysine hydrochloride (Sigma‐Aldrich, cat. no. W384712)
l‐Arginine hydrochloride (Sigma‐Aldrich, cat. no. A92600)
Tris([1‐benzyl‐1*H*‐1,2,3‐triazol‐4‐yl]methyl)amine (TBTA; Sigma‐Aldrich, cat. no. 678937)
*t*‐Butanol (Sigma‐Aldrich, cat. no. 360538)
Copper(II) sulfate (CuSO_4_; Sigma‐Aldrich, cat. no. 451657)
Biotin PEG3 azide (Lumiprobe, cat. no. D3730)
Tris(2‐carboxyethyl phosphine) (TCEP; Sigma‐Aldrich, cat. no. 93284)
Methanol (Fisher Scientific, cat. no. A411‐4), ice cold
Chloroform (Fisher Scientific, cat. no. C607SK‐1), ice cold
Proteomics‐grade urea (Sigma‐Aldrich, cat. no. U5378)
Potassium carbonate (Fisher Scientific, cat. no. P208‐500)
Iodoacetamide (Sigma‐Aldrich, cat. no. 11149)
Sodium dodecyl sulfate (SDS; Sigma‐Aldrich, cat. no. L6026)
Streptavidin agarose resin (Pierce, cat. no. PI20349), pre‐equilibrated with PBS for a few minutes at room temperature
Protein LoBind tubes (Eppendorf or BioPioneer)
Sequencing‐grade modified porcine trypsin and trypsin resuspension buffer (Promega, cat. no. V511)
Formic acid (Sigma‐Aldrich, cat. no. C1016)
C18 Stage Tips (Empore solid‐phase extraction disks, 3M)
Optima‐grade acetonitrile (Fisher Scientific, cat. no. A955‐1)
3‐μm C18 resin (Dr. Maisch DmbH, cat. no. ReproSil‐Pur 120 C18‐AQ)
Polyethyleneimine (PEI) “MAX” (MW 40,000, Polysciences. Inc., cat. no. 24765)
Azide‐rhodamine (Lumiprobe, cat. no. 37130)
4× SDS loading buffer (Life Technologies, cat. no.NP0007)
Polyacrylamide gels (Fisher Scientific, cat. no. XP00125BOX)
Nitrocellulose transfer membrane (Millipore Sigma, cat. no. IPVH00010)

10‐ and 15‐cm tissue culture plates (Fisher Scientific)
6‐well plates (3.5‐cm growth diameter; Fisher Scientific)
Humidified CO_2_ incubator (Forma Series II water‐jacketed CO_2_ incubator, Thermo Scientific)
Incubator (Isotemp Fisher Scientific)
Microcentrifuge (Sorvall Legend Micro 17, Thermo Scientific)
Probe sonicator (Branson Sonifier 250)
Ultracentrifuge (Sorvall MX 120+ micro‐ultracentrifuge, Thermo Scientific)
Microplate reader (BioTek ELx808)
Vortex mixer (Fisher Scientific)
Rotator (Labnet Revolver)
Centrifuge (Sorvall ST 16R, Thermo Scientific)
SpeedVac Concentrator (Savant)
Fused silica capillary tubing (375 μm outer diameter, 75 μm inner diameter; Polymicro Technologies, cat. no. 1068150019)
Laser puller (Sutter)
EASY‐nLC 1200 coupled to an Orbitrap Fusion mass spectrometer (Thermo Scientific)
Raw Xtract (version 1.9.9.2; 2004 release; publicly available at http://fields.scripps.edu/downloads.php) or RawConverter (2015 released; http://fields.scripps.edu/rawconv) software
Integrated Proteomics Pipeline (IP2) software
CIMAGE software (developed in house; publicly available at https://github.com/radusuciu/cimage‐simple)
SDS‐PAGE gel tank (Mini Gel Tank Invitrogen) connected to power source (Bio‐Rad PowerRac Basic Power Supply)
Flatbed fluorescence scanner (Bio‐Rad ChemiDoc MP)

### In situ labeling

RP‐ABPP experiments begin with the treatment of cultured cells with probe, competitor, or probe and competitor mixture followed by proteome harvesting and separation. These steps generate labeled soluble and membrane proteomes at a known concentration from cell culture. The proteomes are then used as the starting material for the rest of the RP‐ABPP experiments. These steps can be modified by type of medium, cell, and probe; thus, SILAC‐specific labeling and preparation steps are included later, in the section on SILAC RP‐ABPP (steps 9‐11).

1 Prepare working stock solutions (∼0.2‐3 M) of probe and competitor in H_2_O with or without 10% DMSO, depending on the solubility of the hydrazinium hydrochloride salt forms of the compounds. Titrate the solutions to pH ∼6.5‐7 with concentrated sodium hydroxide using Colorphast pH indicator strips. Store solutions in aliquots at –80°C.
  These preparations are chemically stable for several months and should be analyzed prior to use to confirm their integrity.
2 Grow low‐passage human embryonic kidney (HEK293T) and human breast cancer (MDA‐MB‐231) adherent cell lines in a humidified 37°C, 5% CO_2_ incubator and expand in DMEM containing high glucose, l‐glutamine, and pyruvate supplemented with 10% (v/v) FBS and 1% penicillin/streptomycin antibiotic/antimycotic.
  In general, gel and MS samples require confluent cells harvested from a 6‐well plate (35 cm growth diameter) and one or two 10‐ or 15‐cm dishes, respectively.
3 Plate and grow cells to near complete confluence at the time of treatment.
4 Wash cells with ice‐cold PBS (pH 7.4) and replenish with serum‐free DMEM (∼15% of normal passage volume, e.g., a 10‐cm plate normally passaged with 10 ml medium would receive 1.5 ml) supplemented with 10 mM Na‐HEPES buffer (pH 7.5). Incubate cells with probe (3 and 1 mM for alkyl and aryl probes, respectively) in the absence or presence of 10‐fold excess non‐clickable analogs (competitors; 30 and 10 mM for propyl and phenyl hydrazine, respectively) for 30 min at 37°C. Premix probe and competitor before coadministering to the cells when applicable.
  Effects of in situ probe treatment on cell viability were previously described (Matthews et al., 2017) to confirm that cells treated with 30 mM compounds for hours beyond the treatment time were stable. No cell death was observed, and the cells remained completely viable, indistinguishable from untreated cells.
5 Wash cells with ice‐cold PBS to remove the probe‐containing medium, harvest by scraping, collect by centrifugation (1400 × *g*, 3 min, 4°C), wash again by resuspension in ice‐cold PBS, and freeze as pellets at –80°C (pellets are stable for months) until ready to proceed with proteome preparation.

### Proteome preparation

6 Resuspend cell pellets on ice in PBS (100‐500 µl) and lyse with a Branson Sonifier probe sonicator (two sets of 6‐10 pulses, 15% duty cycle, output setting 3‐5). Adjust resuspension volume and sonication power appropriately for cell pellet yield.
7 Separate soluble and membrane proteomes by ultracentrifugation for 30‐45 min at 100,000 × *g*, 4°C.
8 Determine protein concentrations for each fraction using the DC protein assay^™^ (similar to the Lowry assay) on a microplate reader.

### SILAC RP‐ABPP

9 Prepare SILAC sample for MS‐based analysis of probe‐labeled proteins:
  - Follow the in situ labeling protocol described in steps 1‐5, passaging each cell line a minimum of six doublings in SILAC DMEM (lysine and arginine free) containing dFBS supplemented with either isotopically enriched l‐[^13^C_6_^15^N_2_]lysine hydrochloride and l‐[^13^C_6_^15^N_4_]arginine hydrochloride or natural‐abundance isotopologues (100 μg/ml each, 550 and 475 μM, respectively). Treat isotopically light cells with non‐clickable analog (at the same concentration of probe in the “enrichment” experiments) or the probe in the presence of 10‐fold excess non‐clickable analog as a competitor (in competition experiments). Treat isotopically heavy cells with the probe for both types of experiments.
    These treatments are performed in non‐SILAC medium, which, we recognize, by introducing natural‐abundance amino acids, could potentially yield false‐negative targets that have very short half‐lives (<30 min).
  - Mix isotopically heavy and light whole cell lysates in equal proportions, and follow the proteome preparation procedure described in steps 6‐8, starting with lysing the cells.
  - Dilute the fractionated equimolar mixture of heavy and light soluble proteomes (∼1‐1.5 mg) to 1 ml in PBS.
  - Add 110 µl of a freshly prepared “click” reagent mixture containing 0.1 mM tris(benzyltriazolylmethyl)amine (TBTA; 60 µl/sample, 1.7 mM in 4:1 DMSO:*t*‐BuOH), 1 mM CuSO_4_ (20 µl/sample, 50 mM in H_2_O), 100 μM biotin‐azide (10 µl/sample, 10 mM in DMSO), and freshly prepared 1 mM tris(2‐carboxyethyl)phosphine (TCEP; 20 µl/sample, 50 mM in PBS or H_2_O) to each sample (1 ml). Vortex mixture and place on a rotator at ambient temperature for 1 hr.
  - Quench the reaction by sequential addition of prechilled methanol (MeOH; 2 ml), chloroform (CHCl_3_, 0.5 ml), and PBS (1 ml) on ice, mixing after each addition.
  - Centrifuge the precipitated proteome 10 min at 5000 × *g*, 4°C, to fractionate the protein interphase from the organic and aqueous solvent layers.
  - Wash the protein pellet with ice‐cold 1:1 (v/v) MeOH:CHCl_3_ (three times, 1 ml each time), and then add ice‐cold 4:1 (v/v) MeOH:CHCl_3_ (2.5 ml) and sonicate (5 pulses, 15% duty cycle, output setting 3‐5) to ensure click reagents are efficiently removed.
  - Pellet the remaining precipitate by centrifugation (5000 × *g*, 10 min, 4°C) and redissolve by mild sonication (3‐5 pulses, 15% duty cycle, output setting 3‐5) in a freshly prepared solution of 6 M proteomics‐grade urea in PBS (500 µl).
  - Reduce disulfides with TCEP (9 mM) pre‐neutralized with potassium carbonate (27 mM) for 30 min at 37°C. TCEP pre‐neutralized with potassium carbonate is freshly prepared by adding 600 mM K_2_CO_3_ to prepared 200 mM TCEP (1: 1, v/v).
  - Alkylate reduced thiols with iodoacetamide (45 mM) for 30 min at ambient temperature protected from light.
  - Add 10% (w/v) SDS stock solution to 2% (w/v) to ensure complete denaturation.
  - Dilute solution to ∼0.2% SDS with PBS (∼5 ml) and incubate on a column containing pre‐equilibrated streptavidin agarose resin (50 μl column volume, 100 µl 1:1 slurry) for ∼1.5‐2 hr at ambient temperature on a rotator.
  - Collect streptavidin beads by centrifugation for 1‐2 min at 1400 × *g*, and wash sequentially, three times each, with 0.2% SDS in PBS (∼10 ml), detergent‐free PBS (∼10 ml), and H_2_O (∼10 ml) to remove unbound protein, excess detergent, and small molecules.
  - Transfer resin to a Protein LoBind tube and digest bound proteins on‐bead overnight at 37°C in ∼200 µl total volume containing sequencing‐grade porcine trypsin (2 μg) in the presence of 2 M urea in PBS) and 1 mM CaCl_2_.
  - Transfer the proteolyzed supernatant to a fresh Protein LoBind tube and acidify with formic acid (5%) to inactivate trypsin. Store at −80°C if needed.
10 Liquid chromatography‐tandem mass spectrometry (LC‐MS/MS):
  Numerous mass spectrometry protocols exist, and historically multidimensional protein identification technology (MudPIT) mass spectrometry protocols have been used for ABPP and RP‐ABPP (Washburn, Wolters, & Yates, 2001). However, we have adapted a one‐dimensional method (substeps a‐g) with a shorter run time for RP‐ABPP (Blaesi et al., 2018).
  a. Desalt the acidified peptide mixture using C18 Stage Tips.
  b. Concentrate desalted samples under reduced pressure in an evacuated centrifuge (SpeedVac) and redissolve in 10 µl of diluent (98% H_2_O, 2% acetonitrile, 0.1% formic acid) for nanoLC‐MS/MS analysis.
  c. Inject a 3‐ to 5‐µl aliquot of this solution in diluent via a nano‐LC system onto a 75‐µm‐inner‐diameter fused‐silica capillary column hand‐packed with C18 resin and with a laser‐pulled tip in solvent A (0.1% formic acid in H_2_O).
  d. Develop the column with a 60‐min gradient of 5%‐100% solvent B (20% H_2_O, 80% acetonitrile, 0.1% formic acid).
  e. Ionize peptides in positive‐ion mode with a flow rate of 300 nl/min and an applied voltage of 2.3 kV.
  f. Collect spectra in a data‐dependent mode such that each scan cycle involves a single high‐resolution (30,000) full MS spectrum of parent ions (MS1 scan from 400 to 1800 *m/z*) collected in the Orbitrap Fusion coupled to a 30 CID‐induced fragmentation (MS2) scans in the ion trap of the 30 most abundant parent ions from the MS1 scan.
  g. Exclude parent ions with unassigned of +1 charge stated by the instrument for fragmentation. Leave all other parameters as default values.
11 Determination of high‐reactivity protein targets:
  - Extract the MS2 spectra for all fragmented parent ions (.ms2 file) from each of the .raw files generated by the instrument (with Xcalibur software) using RAW Xtract or RawConverter with monoisotopic selection (He, Diedrich, Chu, & Yates III, 2015).
  - Search each .ms2 file using the ProLuCID algorithm against a reverse‐concatenated, nonredundant database of the human proteome, and filter using DTASelect 2.0 within the IP2 software.
  - Search cysteine residues with a static modification for carboxyamidomethylation (+57.02146 Da). Search methionine residues with up to one differential modification for oxidation (+15.9949 Da).
  - Require peptides to have at least one tryptic terminus but allow an unlimited number of missed cleavages in the database search. Search each dataset for both light and heavy isotopologues of the same peptide by specifying the mass shift of heavy residues as static modifications on lysine (+8.0142 Da) and arginine (+10.0082 Da) in a coupled “heavy” search.
  - Set the parent ion mass tolerance for a minimum envelope of three isotopic peaks to 50 ppm, the minimum peptide length to six residues, and the false positive rate to 1%, and require the detection of at least two peptides of a protein in order for it to advance to the next step of analysis.
  - Extract and compare heavy and light parent ion chromatograms associated with successfully identified peptides using in‐house software (CIMAGE) as previously described (Weerapana et al., 2010).
  - At least one ion of a coeluting heavy‐light pair must be accurately identified from a fragmentation event that occurred within the retention time window (±10 min) of parent ion elution.
  - Ensure that the correct pair of peaks is quantified by extracting chromatograms within a 10‐ppm error tolerance of the predicted *m/z*, single‐to‐noise ratios >2.5, and “co‐elution correlation scores” and “envelope correlation scores” *R* ^2^ values ≥0.8.
    Peptides detected as “singletons,” where only the heavy ion of a peptide pair is identified, but that pass all other filtering parameters should be given a default assigned ratio of “20,” which is defined as any measured ratio that is ≥20.
  - Determine protein ratios for each replicate by the median peptide ratio derived from three or more unique qualified peptides to further eliminate false positives and stochastic variability in the data.
  - Average protein ratios across ≥3 replicates that comply with these criteria from a single experiment provide final reported ratio values.

### Validation via recombinant expression

12 After obtaining target genes in mammalian expression vectors, grow cells to ∼40% confluence under standard growth conditions, passaging in the appropriate medium.
13 Add the appropriate expression vector (4 µg for 6‐cm dish; control (“mock”) cells should receive an equal amount of the appropriate empty vector) and polyethyleneimine (PEI) “MAX” (MW 40,000) as a transfection reagent under standard transfection conditions [3:1 (w/w) vector/PEI ratio].
14 Incubate cells for ∼48 hr before performing in situ labeling and proteome preparation as described in steps 1‐8.
15 Gel‐based analysis of probe‐labeled proteins:
  - Dilute soluble proteomes from treated cells to 1 mg/ml, 50 µl.
  - Add 6 µl of a freshly prepared “click” reagent mixture containing 0.1 mM TBTA (3 µl/sample, 1.7 mM in 4:1 DMSO:*t*‐BuOH), 1 mM CuSO_4_ (1 µl/sample, 50 mM in H_2_O), 25 μM azide‐rhodamine (1 µl/sample, 1.25 mM in DMSO), and freshly prepared 1 mM TCEP (1 µl/sample, 50 mM in PBS or H_2_O) to each sample (50 µl) to conjugate the fluorophore to probe‐labeled proteins.
  - Immediately mix by vortexing and allow to react at ambient temperature for 1 hr while rotating.
  - Quench the reactions with 4× SDS loading buffer (17 µl).
  - Resolve proteins by SDS‐PAGE (10% acrylamide), loading ∼25 μg total protein per gel lane, and visualize by in‐gel fluorescence scanning on a flatbed fluorescence scanner.
  - Transfer the same gel to nitrocellulose membrane and perform western blotting (as described in Matthews et al., 2017).

## Basic Protocol 2 CHARACTERIZATION OF THE SITE OF PROBE LABELING

The second aim of RP‐ABPP is to determine the site of electrophilic reactivity on each protein target. The peptide labeled is found using isoTOP‐ABPP (Weerapana et al., 2010; Weerapana, Speers, & Cravatt, 2007) experiments, and the specific amino acid residue labeled is found using a hybrid sequencing approach. To identify the peptide labeled, rather than the whole protein, a site‐specific profiling method called isoTOP ABPP is used (Fig. 3B; Weerapana et al., 2007; Weerapana et al., 2010). Following probe treatment of non‐SILAC cells, this method leverages the conjugation to isotopically differentiated, protease‐cleavable biotin‐TEV (tobacco etch virus) tags. After conjugation to heavy and light tags, labeled proteomes are enriched on streptavidin beads and then on‐bead digestion is performed. All unlabeled peptides are then discarded and the remaining labeled peptides are released from the beads through cleavage of the tag (by TEV protease), ultimately generating probe‐labeled peptides as mass‐differentiated pairs. The peptide harboring the labeled residue can be found using the coeluting pair with heavy and light tags (plotted in dark and light green, respectively, in Fig. 3B). Determining which peptide is labeled can give information about where the reactivity is located in the sequence of the protein (e.g., active site, N‐terminus, etc.). These searches enable differentiation between probe‐labeled peptides and other peptides in the sample regardless of identity or mass. To further resolve the site labeled and the mass of the probe‐captured PTM, the MS2 spectra from the isoTOP experiments is assigned via sequencing of the MS2 spectral assignments. The residue(s) containing the modification can be identified, as they will show the known mass shift of the fragmented heavy/light tag, containing covalently bound probe and conjugated biotin‐TEV tag. After all peaks are identified as either an amino acid or amino acid with fragmented tag, the mass of the bound electrophile can be calculated within 1 ppm error. Once the amino acid containing the reactive electrophile has been identified, the next step is to validate and confirm the location by mutating the specific residue. The site is confirmed if mutation of the site enables visualization of a loss of probe labeling. Additionally, expression should be detectable for both the wild‐type and mutant proteins and confirmed via western blot.

Lastly, these results are validated using site‐specific mutagenic analysis based on the loss of in‐gel fluorescence labeling upon alteration of the involved amino acid in conjunction with continued expression of both the wild‐type and mutant proteins (confirmed via western blot). Characterization of the electrophilic site yields information about where the reactivity is located within the greater context of the protein. Determining this site paves the way for a large variety of downstream experiments, including monitoring the electrophile, examining its installation and regulation, and investigating its function. Additionally, one critical output of peptide sequencing is the mass of the probe‐captured electrophile, which is used to determine the PTM structure. IsoTOP‐ABPP, de novo sequencing, and mutagenic analysis protocols are performed as described in Matthews et al. (2017).

### Materials

Light and heavy biotin‐TEV‐azide tag, synthesized in house (Weerapana et al., 2007)
Ac‐TEV protease, 20× TEV buffer, and 0.1 M dithiothreitol (DTT; Invitrogen, cat. no. 12575‐015)
QuikChange II XL site‐directed mutagenesis kit (Agilent, cat. no. 200521)
Custom oligonucleotide primers (IDT)

Additional reagents and equipment for characterizing the site of labeling (Basic Protocol 1)

**Figure 4:**
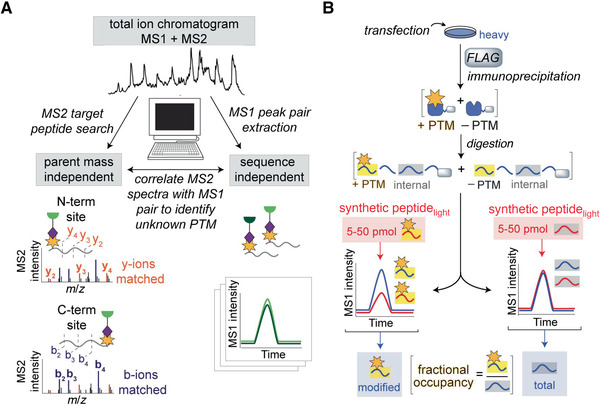
Spectra assignment and stoichiometric measurement of the modified peptide. (**A**) Search strategy to determine the probe‐labeled site from isoTOP ABPP data. To determine the peptide containing the modification, MS1 searches for the coeluting pair with the specified mass difference are performed. To determine the residue labeled, MS2 spectra are assigned by de novo sequencing. (**B**) Scheme of stoichiometry measurement for modified peptide. Using a known quantity of synthetic standards of modified and total protein allows the calculation of fractional occupancy.

1 IsoTOP ABPP sample preparation to isolate probe‐captured peptides:
  - After in situ labeling, treat wild‐type or transfected cells with probe and process the proteome as described in Basic Protocol 1, steps 4‐8. Dilute soluble proteomes (2 mg total protein) to 1 ml in PBS.
  - Conjugate half of the proteome (0.5 ml) to the light TEV tag and the other half to the heavy TEV tag. Scale click reactions to maintain final concentrations of 0.1 mM TBTA, 1 mM CUSO_4_, 100 μM of 5 mM light or heavy biotin‐TEV‐azide in DMSO), and 1 mM TCEP. Vortex the mixture and place on a rotator at ambient temperature for 1 h.
  - Centrifuge samples 5 min at 16,000 × *g*, 4°C, add ice‐cold methanol (0.5 ml), and mildly sonicate (3‐5 pulses, 15% duty cycle, output setting 3‐5) resulting pellets.
  - Combine the light‐ and heavy‐labeled proteomes and centrifuge once more.
  - Solubilize proteomes with 1.2% SDS (1 ml in PBS), using sonication to aid dissolution.
    Store at –80°C overnight if desired.
  - Dilute samples to ∼0.2% SDS with PBS (∼5 ml) and incubate with pre‐equilibrated streptavidin agarose resin (100 µl 1:1 slurry) for ∼2‐3 hr at ambient temperature while rotating.
  - Wash resin as described above in Basic Protocol 1, step 9(m), transfer to fresh microcentrifuge tubes, and resuspend in 6 M urea in PBS (500 µl).
  - Reduce and alkylate cysteines with TCEP and iodoacetamide, respectively, as described in Basic Protocol 1, step 9(i) and (j).
  - Wash resin once with PBS to remove the reagents, and digest bound proteins with trypsin (2 μg) for 8‐12 hr at 37°C in the presence of 2 M urea in PBS (200 µl) and 2 mM CaCl_2_.
  - Remove unmodified peptides, urea, and trypsin by sequential washes with PBS (five times, 0.5 ml each ) and H_2_O (five times, 0.5 ml) each.
  - Transfer the resin to fresh tubes and equilibrate with TEV buffer (50 mM Tris, pH 8).
  - Release remaining immobilized peptides with ∼1‐2 μM TEV protease in ∼200 µl TEV buffer at 30°C for 3‐5 hr while shaking gently.
  - Collect and recover heavy‐ and light‐labeled peptides from the resin by elution with H_2_O (two washes, 50 µl each).
  - Desalt the samples using C18 stage tips.
    Store samples at –80°C and analyze as soon as possible or within several days. This is important because the chemical stability of the adducts cannot be anticipated given that the structures may not yet be known.
2 Characterization of probe‐labeled peptides by isoTOP ABPP:
  - Collect proteomics data as described in Basic Protocol 1, step 10. Search data on the MS1 level for paired spectra of coeluting peaks (shown at right in Fig. 4A). Search every recorded monoisotopic precursor mass 6.0138 Da (±5 ppm) upstream and downstream in each total ion (MS1) spectrum for a possible isotopic partner, taking into account +2 and +3 charge state differences (3.0069 and 2.0046 Da, respectively). Require that the relative intensity of the monoisotopic peak be ≥5% of the base peak of each spectrum, that there be at least three peaks in each isotope profile (envelope), and that the Euclidean distance between the two isotope profiles be ≤0.2.
    This in‐house‐generated script is now publicly available to download from GitHub at https://github.com/matthewslab/probe.
  - Group pairs with the same *m/z* values (±5 ppm) and retention times to eliminate duplicates.
  - Analyze pairs of parent ions from transfected versus mock‐transfected cells and from three biological replicates.
    This search is valuable because it allows all probe‐labeled peptides, regardless of their mass or identity, to be distinguished from other ions in the sample through their unique pair feature with a defined mass differential.

### De novo sequencing

3 Assign the MS2 spectra manually (depicted at left in Fig 4A). Modify instrument method such that both the parent and fragment ions are measured in the Orbitrap Fusion instrument to gain high‐resolution data for both (resolutions for MS1 and MS2 were set at 30,000 and 7500, respectively) to be certain of the peak assignments and charge state of the tag‐specific b‐ions. Select only the five most abundant parent ions for fragmentation per cycle (versus 30 if the spectra are collected in the ion trap) to account for increased scan time. Extend the number of MS2 spectra collected per cycle to ten if the data are generated on the Orbitrap.
4 Search the MS2 spectra for diagnostic ions of the peptide. Find highly abundant fragments that represent diagnostic markers for the unmodified portion of the peptide and other fragmentation products representing the portion that contains the modification and that is differentially labeled by the isotopic tags. Compare the spectra for the heavy and light peptides and observe the appropriate ion shift appropriately (6.0138 Da).
5 Use RawConverter to extract accurate monoisotopic *m/z* values for each MS2 spectra. Downstream structure determination will be based on the correct precursor mass calculated from the residue containing the fragmented tag, as further described in Basic Protocol 3.

### Mutagenic analysis

6 Generate mutants with primers containing the desired mutations and their respective complements.
7 Transfect cells as described above in validation via recombinant expression.
8 Label and prepare proteomes as described above in in situ labeling and proteome preparation sections.
9 Conjugate to fluorophore, visualize via in‐gel fluorescence, and perform western blotting as described above in Basic Protocol 1, step 15.
  The site is confirmed if mutation of the site results in visualization of a loss of probe labeling while expression of both the wild‐type and mutant proteins remains present (confirmed via western blot).

## Basic Protocol 3 DETERMINATION AND QUANTITATION OF ELECTROPHILE STRUCTURE

The third and final goal of RP‐ABPP is to determine the structure of the electrophile PTM. Initially, a structure match is proposed using the mass information about the probe‐captured PTM from de novo sequencing and chemical intuition about the residue and probe chemical reactivity. Information about enzyme class or similar motifs may also be useful. Here, peptides are coeluted with synthetic standards to (1) confirm the proposed structure, as well as providing another confirmation of the site of labeling, and (2) quantify the fraction of protein bearing the modification inside the cell. Peptide coelution protocol is performed as described (Matthews et al., 2017). Validation of electrophilic site and structure utilizes coelution of isotopically differentiated samples, light synthesized peptide with proposed probe‐captured electrophile PTM, and probe‐labeled heavy SILAC cells. The fraction of modified protein can be determined by coeluting (light) synthetic modified and internal standards with (heavy) endogenous modified and internal peptides as depicted in Figure 4B. In these experiments, the internal peptide represents the total protein in the cell (both modified and unmodified protein). The absolute quantities of both the endogenous modified and internal peptides can be calculated using the known amounts of the respective standards added to the sample and the relative peak areas of the standard to the endogenous peptides, as shown by fractional occupancy in Figure 4B. This is the only protocol in RP‐ABPP that does not utilize probes, and as such, downstream purification relies on other means, such as FLAG immunoprecipitation.

### Materials

Tris•Cl, pH 8 (Sigma‐Aldrich, cat. no. T6664)
Dithiothreitol (DTT; Fisher Scientific, cat. no. 242284
Trifluoroacetic acid (TFA; Sigma‐Aldrich, cat. no. 302031)
Aniline (Sigma‐Aldrich, cat no. 242284)
Copper(II) chloride (CuCl_2_; Fisher Scientific, cat. no. C455‐500)

Reagents and equipment for peptide coelution (see Basic Protocols 1 and 2)

### Validation of proposed structure by coelution with synthetic standard

1 Prepare synthetic standard:
  - Synthesize SCRN3 probe–captured glyoxylyl tryptic peptide using solid‐phase peptide synthesis as previously described (Matthews et al., 2017), including the proposed electrophilic PTM.
  - Dilute stocks of the heavy and light biotin‐azide tags (0.25 μmol of each) to 250 μM with 1 ml 50 mM Tris•Cl, pH 8, supplemented with 1 mM DTT in the presence of 0.4 μM TEV protease. Incubate the reaction overnight at 30°C.
  - Concentrate the reaction to ∼200 µl and precipitate the protease with an equal volume of acetonitrile and pellet.
  - Purify the supernatants containing the products by reverse‐phase high‐performance liquid chromatography (RP‐HPLC; gradient: 0%‐70% 90:10 (v/v) acetonitrile/H_2_O in H_2_O, 0.05% (v/v) TFA in 35 min; flow rate: 1 ml/min) using a Phenomenex Jupiter 5 μm C18 300 Å (150 × 4.6 mm) column.
    ESI‐MS calc'd for C_15_H_29_N_8_O_4_^+^ and ^13^C_5_C_10_H_29_^15^NN_7_O_4_^+^ ([M+H]^+^): 385.2 and 392.2, found 385.0 and 391.2, respectively, for SI‐10_light_ and SI‐10_heavy_. The yield of the reaction was low, as major peaks for the uncleaved reactants remained.
  - Conjugate hydrazone alkyne peptide to cleaved heavy and light tags via Cu(I)‐catalyzed azide‐alkyne cycloaddition. Add TBTA (0.1 mM), CuCl_2_ (1 mM), and TCEP (1 mM) to a 0.5‐ml mixture of the hydrazone alkyne peptide (∼0.3 mM, ∼0.15 μmol) and either form of cleaved tag (∼60 μM, ∼0.03 μmol of each) in sodium phosphate buffer (60 mM, pH 7).
  - Incubate reactions at ambient temperature for ∼2 hr and purify by RP‐HPLC (gradient: 5%‐55% 90:10 (v/v) acetonitrile/H_2_O in H_2_O, 0.05% (v/v) TFA in 25 min; flow rate: 1 ml/min) using a Phenomenex Jupiter 5μm C18 300 Å (150 × 4.6 mm) column.
    For the glyoxylyl hydrazine standard, ESI‐MS calc'd for C95H146N29O28+ and 13C5C90H14615NN28O28+ ([M+H]+): 2141.7 and 2147.1, found 2141.8 and 2147.6, respectively, for light and heavy isotopologues. The estimated yields of the final standards (SI‐11heavy and SI‐11light) were ∼1 nmol as calculated based on the measured molar absorptivity (e340) of 26,036 M–1cm–1 for SI‐8. This metric compares favorably with that for other hydrazone species.
  - Neutralize the product with sodium phosphate buffer (25 mM, pH 7), lyophilize, and freeze in aliquots (5‐50 pmol each) at –80°C (product is stable for months).
2 Coelute with synthetic standard:
  - Isolate tagged peptide pairs of protein labeled with probe as described in Basic Protocol 2, step 1, except growing cells in standard SILAC medium.
  - Analyze the sample in the absence and presence of 0.5 pmol of natural‐abundance synthetic standards also modified with probe and conjugated with the tags.
  - Dilute standard in water, verify concentration spectrophotometrically, and add 0.5 pmol to the digested sample just before loading the column for analysis.

### Determination of absolute stoichiometry with synthetic isotopologues

3 Transfect cells grown in heavy SILAC medium using a plasmid containing genes for the protein and FLAG.
4 Lyse cells in PBS (pH 7.4) and fractionate by ultracentrifugation for 30‐40 min at 100,000 × *g*, 4°C.
5 Dilute the sample to 1 ml with 50 mM Na‐HEPES buffer (pH 7.5) supplemented with 500 mM NaCl and 1% Triton X‐100.
6 Pellet unsolubilized protein remaining in the sample by centrifugation, denature with a small volume 10% SDS for 1 hr at 37°C, and then recombine with the diluted reaction, ensuring that the final concentration of SDS does not exceed ∼0.2%.
7 Incubate the completely resolubilized sample with anti‐FLAG resin overnight at 4°C by rotation.
8 Wash resin by resuspension and centrifugation with the same buffer but supplemented only with 500 mM NaCl (5 × 1 ml) followed by only 100 mM NaCl (2 × 1 ml).
9 Elute the bound protein by incubating the beads in PBS containing 8 M urea (twice, 50 µl each time) for ∼1 hr at 37°C.
10 Reduce cysteines with 10 mM TCEP (pre‐neutralized with 30 mM potassium carbonate) for 30 min at 37°C, and then alkylate with 20 mM iodoacetamide under the same conditions but protected from light.
11 Dilute the samples to 2 M urea with PBS, add with 2 μg trypsin supplemented with 1 mM CaCl_2_, and incubate overnight at 37°C to allow trypsin digestion.
12 Inactivate trypsin with 5% formic acid.
13 Dilute the natural‐abundance probe–labeled peptide standard in PBS.
14 Dope the digested protein sample with the modified standard, as well as an internal peptide that represents the total protein (5‐50 pmol of each), just before analysis by the same method used for proteomic profiling. Adjust absolute amounts of standards to achieve nearly comparable peak intensities for quantitation.

## REAGENTS AND SOLUTIONS

### 1.7 mM TBTA in 4:1 t‐butanol/DMSO

Prepare 200 µl of 1.7 mM TBTA solution in DMSO and mix thoroughly. Add 800 µl *t*‐butanol. Can be stored at room temperature for months; prepare fresh solution if a precipitate is observed.

t‐Butanol is included to aid “click” reaction, but freezes at room temperature by itself; including the DMSO prevents freezing. TBTA is added to reduce the number of solutions involved.

## COMMENTARY

### Background Information

PTMs play a critical role in protein function and regulation diversity, as well as disease incidence. Therefore, elucidation of PTM characteristics, installation machinery, and biological impact is fundamental in understanding diseases and, consequently, proposing diagnostic biomarkers and therapeutic targets. To study highly modified, diverse, functionally rich proteins, they must be examined in the cell type and state where they are active. One way to do so is using probes to target functionality in the proteome, as is done in ABPP and RP‐ABPP. Thus, activity can be evaluated independently of expression.

ABPP has been used as a tool for enzyme, inhibitor, and drug discovery. In ABPP, nucleophilic sites on proteins can be predicted from sequence because specific amino acids (e.g., serine, cysteine, lysine, tyrosine, and aspartate) can be exploited as nucleophiles in enzyme active sites, whereas in RP‐ABPP, electrophiles cannot be predicted based on sequence. By contrast to intrinsic nucleophiles, electrophiles are absent among common amino acids and must be installed post‐translationally. The prediction in ABPP allows profiling of anticipated activity at the predicted site on protein, whereas RP‐ABPP experiments must begin by discovering proteins harboring electrophilic modification, followed by identifying the site of modification and characterizing the structure of the electrophile, because there is no prediction to rely upon and small‐molecule nucleophiles remain underdeveloped with respect to their proteome‐wide reactivity toward electrophilic PTMs. Thus, in addition to the applications of ABPP, RP‐ABPP can also be applied for de novo discovery. As RP‐ABPP is the first and only global, unbiased approach to identify electrophilic PTMs, the majority of functional electrophiles in proteins were discovered on a case‐by‐case basis by investigating the in vitro biochemical reactions catalyzed by purified enzymes.

Diverse electrophilic modifications are well known to confer functions and are usually acquired through covalent installation (Klinman & Bonnot, 2014; Okeley & Van Der Donk, 2000) or exogenous cofactor binding (Phillips, 2015). Proteins have been known to incorporate more than ten classes of electrophiles for essential functions, including catalysis (Walsh, 2006). PTMs of this type are generally referred to as cofactors, meaning essential chemical machinery that is required for catalysis and is generally regenerated in each catalytic cycle. In other words, a cofactor can act as a reactive chemical switch that turn enzyme activity on or off, similar to the role of other important PTMs such as methylation and phosphorylation in signal transduction. The key difference, in terms of detection, is that the latter class of PTMs are chemically stable, and thus readily detectable and quantifiable without any chemical trapping or derivatization. In contrast, functional electrophilic cofactors are reactive and transient, generally cannot be predicted by sequence, and escape detection by untargeted proteomics. An additional challenge of examining protein electrophiles is that PTMs are, by nature, context dependent. Because of their dynamic regulation that depends on cell type, cell state and a variety of environmental stimuli, these chemically reactive PTMs stabilized and contained within protected active sites must be captured in their native state in order to be studied in a manner that is compatible with general proteomics protocols.

### Critical Parameters and Troubleshooting

The results generated from target identification and quantification (Basic Protocol 1) are quite robust. When RP‐ABPP was performed in two cell lines with two probes, ∼40 high‐reactivity targets (average protein enrichment ratio ≥5 and competition ratio ≥4) were identified, only two of which were previously known to harbor an electrophile (Matthews et al., 2017). Given the promise of these initial results, which suggest that there are likely many more biologically important electrophilic or electron‐deficient functionalities to discover, performing identification and quantification with other probes and in other cell lines is likely to greatly expand our knowledge of known functional electrophiles. The experiments involved in target identification and quantification are both diverse and essential. Before any downstream experiments or analysis can be performed, targets must be identified. Most RP‐ABPP experiments build upon each other. For example, isoTOP ABPP experiments (Basic Protocol 2) start with in situ labeling and proteome preparation, and parts of MS‐based analysis are repeated as well. Additionally, mutagenic analysis relies upon gel‐based analysis and also begins with in situ labeling and proteome preparation.

The isoTOP ABPP sample preparation is particularly laborious and requires extensive planning as the samples are very sensitive and should be analyzed quickly after performing the preparation. Furthermore, as is the case with identification and quantification, incubation and washes of the samples with streptavidin resin is a critical step. Excess urea not removed during washes can impact TEV protease activity, thereby causing difficulty in releasing immobilized peptides (Weerapana et al., 2007). Further evidence of this issue is that two common sources of problems in these experiments are (1) incomplete trypsin digestion and (2) incomplete TEV digestion (Weerapana et al., 2007). Urea is used as a denaturing agent in these experiments, but high urea concentrations can reduce trypsin activity, and TEV protease is sensitive to even trace amounts of urea; as such, these steps represent critical points in this method (Weerapana et al., 2007).

The robustness of this alternative search strategy (laid out in Fig. 4A) relates to the fact that to make the determination, in the MS1 and MS2 searches, no sequence information and no parent mass information are required, respectively. This approach is unlike nearly all other peptide identification platforms because they are sequence based. Historically, to identify a modified peptide, the modified mass of the amino acid and the amino acid being modified must be predicted. Therefore, our search strategy allows true de novo discovery of chemically reactive PTMs that cannot be predicted in any way. Once the site has been determined and mutations at the electrophilic site have been generated, this allows extensive further experimentation. In downstream experiments, the wild‐type protein harboring the modification and the mutant lacking said modification can be used to interrogate installation, function, and other aspects of the modification.

As discussed in the introduction, PTMs are generally context dependent, and because of different regulation and demands of the cells, generally not all proteins in the proteome are modified. Using the coelution protocol (Basic Protocol 3) to determine the fraction of the protein containing the modification can be utilized in further experiments to examine this context dependency and assess what portion of the protein is modified in different settings and how its installation is regulated by the cell. RP‐ABPP has been used for de novo discovery of a previously unknown modification (Matthews et al., 2017) suggesting that other human proteins might harbor new electrophilic modifications as well. As such, electrophilic structure determination experiments could yield novel and far‐reaching results. RP‐ABPP has many potential future applications. Here, these experiments are limited to cell culture; however, they can easily be adapted for use in bacteria, pathogens, tissue, or living animals. This method potentially opens up a new area, another hemisphere of the reactive proteome, the “electrophilome.” Based on targets generated from only two probes in two human cell lines (Matthews et al., 2017), there are more electrophile‐bound proteins to be discovered and disease relationships to be explored. In addition, the tools of this platform can also be used to further investigate various aspects of the proteins and electrophilic modifications discovered: for example, installation mechanisms, functions of these electrophiles, and potential connections with metabolic regulation. Additionally, as many of the protein targets discovered have strong associations to poorly understood disease mechanisms (e.g., APP in Alzheimer's disease and fat mass and obesity‐associated protein [FTO] in obesity), the installation and function of these modifications could play important roles in, or yield information about, the pathogenesis of these diseases. Lastly, given hydrazines’ ability to covalently inhibit enzymes through reaction with their electrophilic cofactors, these experiments could ultimately be used in inhibitor discovery and to launch pharmaceutical trials.

### Understanding Results

Electrophilic modifications are not easily predicted from protein sequence, and sequence predictions do not yield information about function or activity. Initial results from RP‐ABPP yielded protein targets that were not previously known to harbor electrophiles. These targets with known or as‐yet‐unknown functionality are strongly implicated in diseases such as cancer, Alzheimer's disease, and obesity (Matthews et al., 2017). RP‐ABPP methodology also discovered a novel cysteine‐to‐glyoxylyl transformation in the protein SCRN3, an uncharacterized protein predicted by sequence to possess opposite reactivity, a nucleophilic thiol involved in hydrolase activity (Matthews et al., 2017). In each replicate for SCRN3, the top two most abundant pairs, based on direct mass ion intensity measurements, were tryptic peptides of the SCRN3 N‐terminus that matched the masses for probe 2‐hydrazone products of glyoxylyl and pyruvoyl N‐terminal modifications. These pairs were the only two *m/z* species observed across all three replicates and were not identified in samples (two biological replicates) from mock‐transfected cells, highlighting that their detection was robust and specific to SCRN3 transfection.

**Figure 5:**
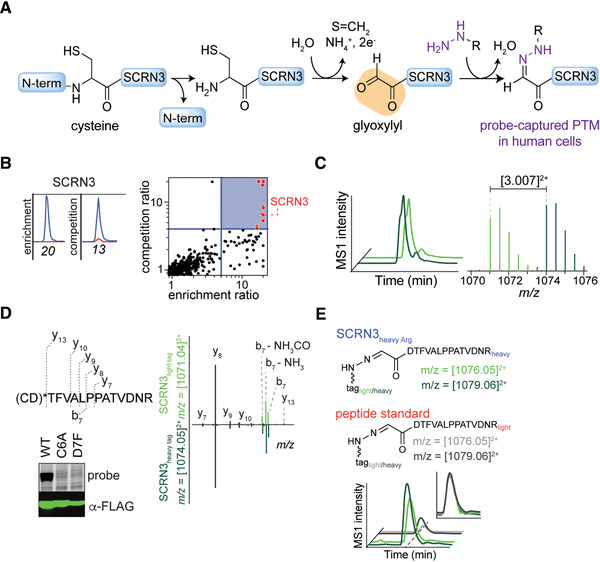
Sample data from RP‐ABPP experiments. Adapted from published work (Matthews et al., 2017). (**A**) Reaction scheme of probe captured glyoxylyl on SCRN3. (**B**) Extracted parent ion chromatograms and corresponding H/L ratios for tryptic peptides of the SCRN3 protein of probe quantified in enrichment and competition (left) and quadrant plot of average competition versus enrichment SILAC ratios from quantitative proteomics experiments (right). (**C**) Extracted MS1 ion chromatograms (left) and corresponding isotopic envelopes (right) for coeluting heavy‐ and light‐tagged peptides labeled by probe (in dark and light green, respectively). (**D**) Comparison of high‐resolution MS2 spectra generated from light‐ versus heavy‐tagged parent ions. The y‐ions resolve the modified site (*) to the N‐terminal cysteine and/or adjacent aspartate. Probe‐labeling and expression profiles of Cys6‐to‐Ala6 (C6A) and Asp7‐to‐Phe7 (D7F) mutant SCRN3 proteins compared to wild‐type (WT) SCRN3. (**E**) Heavy‐Arg/Lys‐labeled SCRN3‐transfected cells treated with probe, and then processed by isoTOP‐ABPP, furnishes an isotopically differentiated probe‐labeled SCRN3 peptide pair (light and dark green), which coelutes with a light‐amino‐acid‐labeled probe–glyoxylyl6‐Arg20 standard (also an isotopically differentiated peptide pair; light and dark gray). Inset chromatogram shows all four traces scaled to the same intensity (inset plot) to show coelution of endogenous and standard probe–glyoxylyl6‐Arg20 SCRN3 peptides.

The results of the RP‐ABPP experiments used to discover the glyoxylyl group are depicted in Figure 5, showing the reaction of the probe with the glyoxylyl group to form a stable hydrazone (Fig. 5A). Enrichment and competition experiments revealed high‐reactivity targets, red dots in the upper right blue quadrant in Figure 5B, including SCRN3 (Matthews et al., 2017). As shown in Figure 5C, isoTOP ABPP extracted MS1 ion chromatograms (left) and isotopic envelopes (right) demonstrate coelution and specific mass differentiation of the labeled SCRN3 peptide (Matthews et al., 2017). The labeled residue was determined by de novo sequencing and validated via mutagenic analysis (Fig. 5D). The y‐ions resolve the modified site to the N‐terminal cysteine and/or adjacent aspartate, and mutation profiles of Cys6‐to‐Ala6 (C6A) and Asp7‐to‐Phe7 (D7F) show a lack of probe labeling compared to wild‐type (WT) SCRN3, suggesting that both Cys6 and Asp7 must be present for labeling to occur (Matthews et al., 2017). Lastly, as shown in Figure 5E, the glyoxylyl structure and site were confirmed by coelution with synthetic standards (Matthews et al., 2017). The discovery of the glyoxylyl demonstrates the utility of RP‐ABPP in discovering previously unknown electrophilic modifications, pointing to the possibility of other electrophilic functionality in the proteome. As such, RP‐ABPP is an incredibly useful, versatile, and necessary method, being the only global unbiased approach to discover electrophilic cofactors.

### Time Consideration

If all materials are on hand, including near‐confluent, isotopically differentiated cells, identification and quantification of probe‐reactive proteins (Basic Protocol 1) will likely take ∼2‐3 days. Characterization of the site of probe labeling (Basic Protocol 2) should take 4‐5 days, or potentially longer depending on method of mutant generation. Lastly, electrophile structure determination and quantitation (Basic Protocol 3) will take 2‐3 days.

### Author Contributions

**Suzanne E. Dettling** Conceptualization; data curation; investigation; methodology; project administration; validation; writing‐original draft; writing‐review & editing. **Mina Ahmadi** Data curation; methodology; validation; writing‐original draft; writing‐review & editing. **Zongtao Lin** Data curation; methodology; project administration; validation; writing‐review & editing. **Lin He** Data curation; methodology; validation; writing‐review & editing. **Lin He**: Data curation; methodology; validation; writing‐review & editing. **Megan L. Matthews** Conceptualization; data curation; formal analysis; funding acquisition; investigation; methodology; project administration; resources; software; supervision; validation; visualization; writing‐original draft; writing‐review & editing.

### Conflicts of Interest Statement

M.L.M. is a founder of Zenagem, LLC.
